# Supplementary material for: Primary exposure to SARS-CoV-2 variants elicits convergent epitope specificities, immunoglobulin V gene usage and public B cell clones
Source: Nat Commun. 2022 Dec 14;13:7733. doi: 10.1038/s41467-022-35456-2 (PMC9748393; doi:10.1038/s41467-022-35456-2)
Supplement: Supplementary file 1 — Supplementary Information [file 41467_2022_35456_MOESM1_ESM.pdf]

Primary exposure to SARS-CoV-2 variants elicits convergent epitope specificities, immunoglobulin V gene usage and public B cell clones

Noemia S. Lima[1*], Maryam Musayev[1*], Timothy S. Johnston[1*], Danielle A. Wagner[1*], Amy R. Henry[1], Lingshu Wang[1], Eun Sung Yang[1], Yi Zhang[1], Kevina Birungi[1], Walker P. Black[1], Sijy O'Dell[1], Stephen D. Schmidt[1], Damee Moon[1], Cynthia G. Lorang[1], Bingchun Zhao[1], Man Chen[1], Kristin L. Boswell[1], Jesmine Roberts-Torres[1], Rachel L. Davis[1], Lowrey Peyton[1], Sandeep R. Narpala[1], Sarah O'Connell[1], Leonid Serebryannyy[1], Jennifer Wang[1], Alexander Schrager[1], Chloe Adrienna Talana[1], Geoffrey Shimberg[1], Kwanyee Leung[1], Wei Shi[1], Rawan Khashab[2], Asaf Biber[2,3], Tal Zilberman[2,3], Joshua Rhein[4], Sara Vetter[5], Afeefa Ahmed[4], Laura Novik[1], Alicia Widge[1], Ingelise Gordon[1], Mercy Guech[1], I-Ting Teng[1], Emily Phung[1], Tracy J. Ruckwardt[1], Amarendra Pegu[1], John Misasi[1], Nicole A. Doria-Rose[1], Martin Gaudinski[1], Richard A. Koup[1], Peter D. Kwong[1], Adrian B. McDermott[1], Sharon Amit[6], Timothy W. Schacker[4], Itzchak Levy[2,3], John R. Mascola[1], Nancy J. Sullivan[1], Chaim A. Schramm[1#], Daniel C. Douek[1#]

1. Vaccine Research Center, National Institute of Allergy and Infectious Diseases, National Institutes of Health. Bethesda, MD 20892, USA.
2. Infectious Disease Unit, Sheba Medical Center, Ramat Gan 5262112, Israel.
3. Sackler Medical School, Tel Aviv University, Tel Aviv 6997801, Israel.
4. Department of Medicine, University of Minnesota Medical School, Minneapolis, MN 55455, USA.
5. Minnesota Department of Health, St Paul, MN 55164, USA
6. Clinical Microbiology, Sheba Medical Center, Ramat-Gan 5262112, Israel.

*equal contribution
#correspondence to chaim.schramm@nih.gov and ddouek@nih.gov

Table of Contents

1. **Supplementary Figures**
   Figure S1: Additional serology and epitope mapping data 2
   Figure S2: Gating strategy to assess T cell responses 3
   Figure S3: Rapid assembly, transfection and production of immunoglobulin (RATP-Ig) workflow 4
   Figure S4: Antigen-specific B cell sorting 5
   Figure S5: Validation of RATP-Ig screening with synthesized plasmids 6
   Figure S6: Clonal expansion in each individual 7
   Figure S7: SARS-CoV-2-specific light chain V gene usage frequencies 8

2. **Supplementary Tables**
   Table S1: Details of the study cohort 9
   Table S2: Sample recovery from 10x Genomics 10
   Table S3: Significant differences in gene-usage 11
   Table S4: Antibodies information 12
   Table S5: Primer sequences for RATP-Ig and Ig sequencing 13

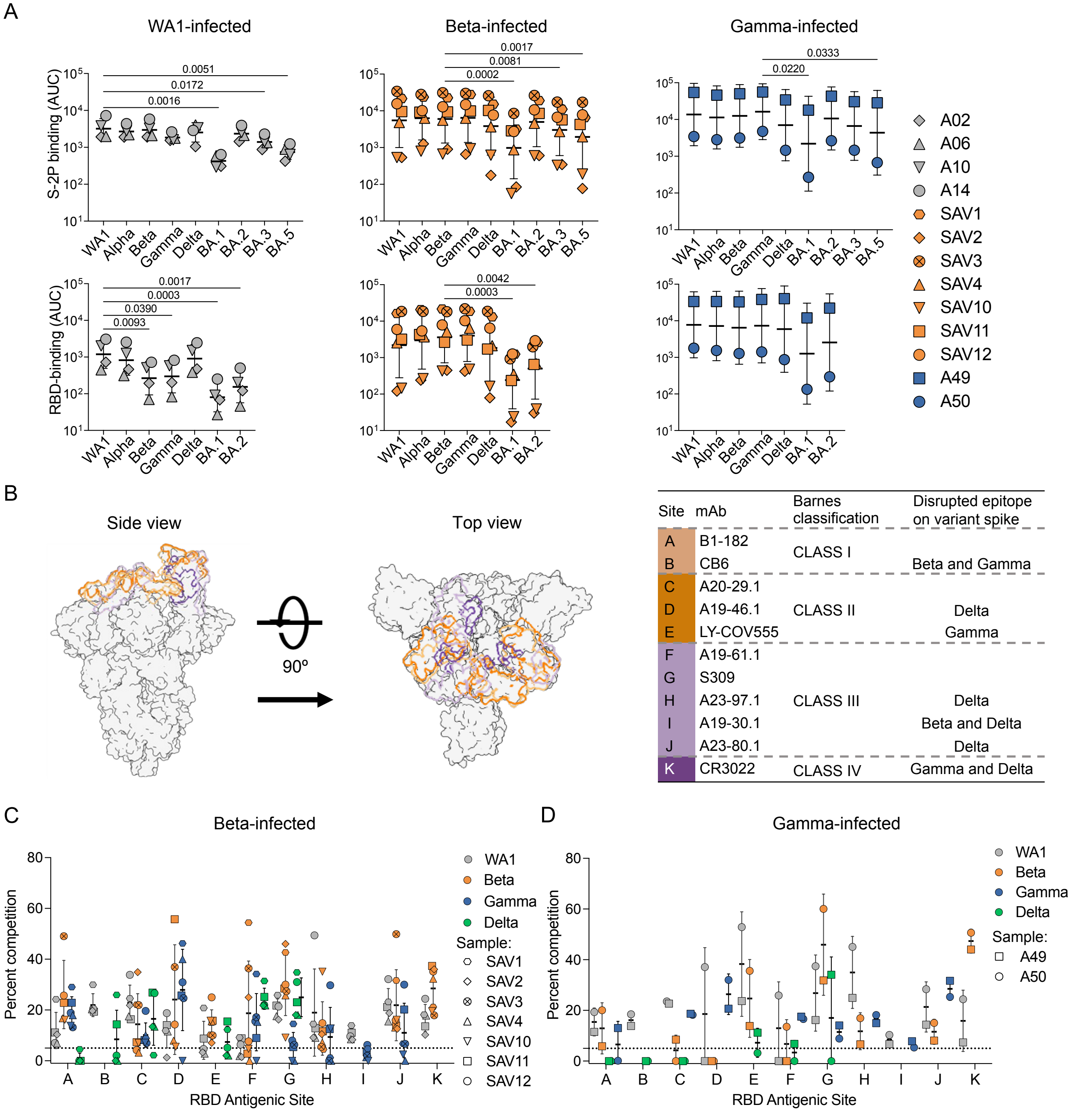

Site	mAb	Barnes classification	Disrupted epitope on variant spike
A	B1-182	CLASS I	
B	CB6		Beta and Gamma
C	A20-29.1		
D	A19-46.1	CLASS II	Delta
E	LY-COV555		Gamma
F	A19-61.1		
G	S309		
H	A23-97.1	CLASS III	Delta
I	A19-30.1		Beta and Delta
J	A23-80.1		Delta
K	CR3022	CLASS IV	Gamma and Delta

Supplementary Fig. 1: Additional serology and epitope mapping data. (**A**) Binding antibody titers to S-2P spike (top panels) and RBD (bottom panels) from different variants indicated on the x-axis. Symbol shapes indicate each donor as shown on the right (more information about the donors on Supplementary Table 1). Bars indicate geometric means with geometric standard deviations. Statistical significance was determined by Friedman test with a two-step linear step-up procedure of Benjamini, Krieger and Yekutieli. (**B**) Structural schematic of spike protein showing epitopes from monoclonal antibodies used for RBD epitope mapping by competition assay. (**C**) Epitope mapping of Beta-infected individuals on WA1, Beta, Gamma and Delta spike proteins. Competition cannot be measured for disrupted epitopes indicated on panel B, and therefore symbols are missing at these sites. Bars indicate means with standard deviations. Measurements for each individual were performed in duplicate and averaged; (**D**) Epitope mapping of Gamma-infected individuals on WA1, Beta, Gamma and Delta spike proteins. Competition cannot be measured for disrupted epitopes indicated on panel B, and therefore symbols are missing at these sites. Bars indicate means with standard deviations.

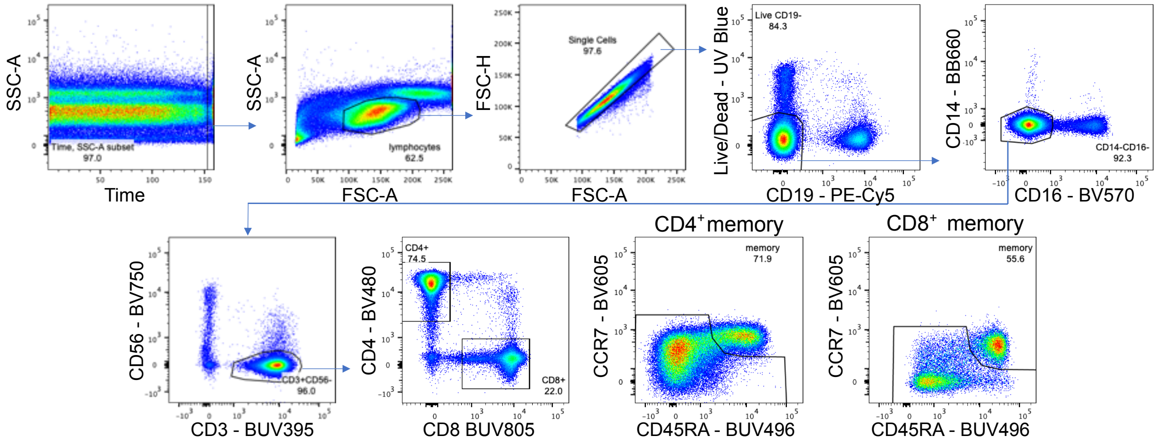

Supplementary Fig. 2: Gating strategy to assess T cell responses by flow cytometry from CD4+ and CD8+ T cell memory populations.

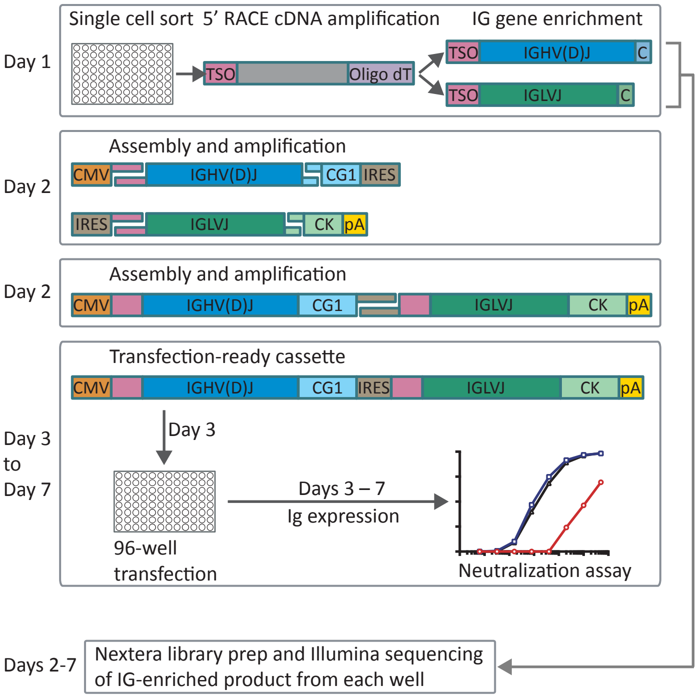

Supplementary Fig. 3: Rapid assembly, transfection and production of immunoglobulin (RATP-Ig) workflow. 5’-RACE is used to generate total cDNA. Full-length heavy and light chain immunoglobulin V genes are enriched by PCR and assembled into recombinant mAb linear expression cassettes. In parallel, V gene libraries are synthesized and sequenced by NGS. Final cassettes are transfected into 96-well Expi293 microtiter cultures, and culture supernatants are collected up to 7 days after initial sort for functional screening.

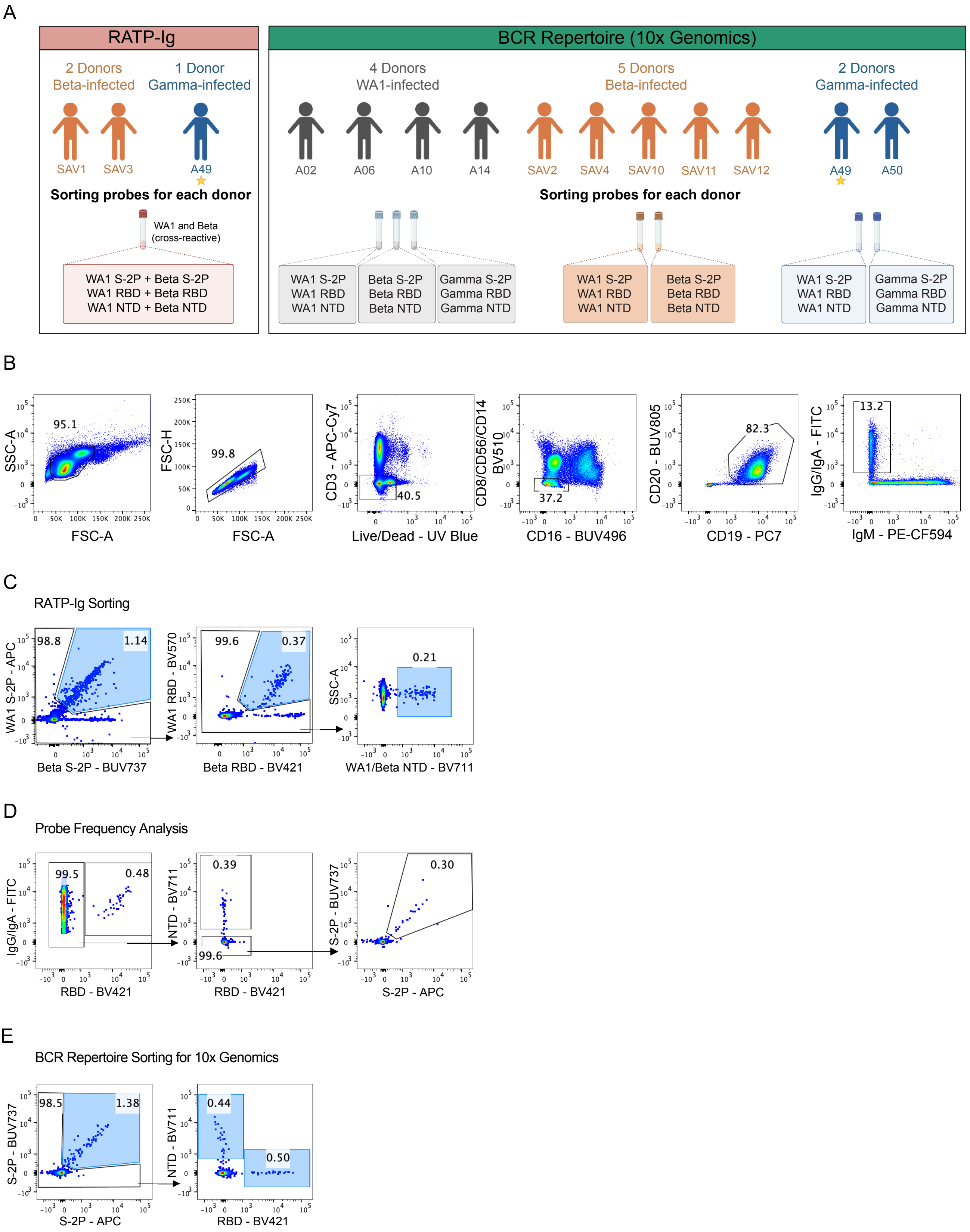

Supplementary Fig. 4: Antigen-specific B cell sorting. (**A**) Schematic showing infection history and sorting strategy for each sequencing approach (RATP-Ig or BCR repertoire by 10x Genomics). The individual marked with a star was used for both RATP-Ig and total BCR repertoire sequencing. (**B**) Flow cytometry representative plots and gating strategy for class-switched memory B cells. (**C**)-(**E**) Representative plots and gating strategy for sorting and analysis of antigen-specific cells for (**C**) RATP-Ig, (**D**) Frequency analysis, and (**E**) Repertoire sequencing. Final sort gates are shown in blue.

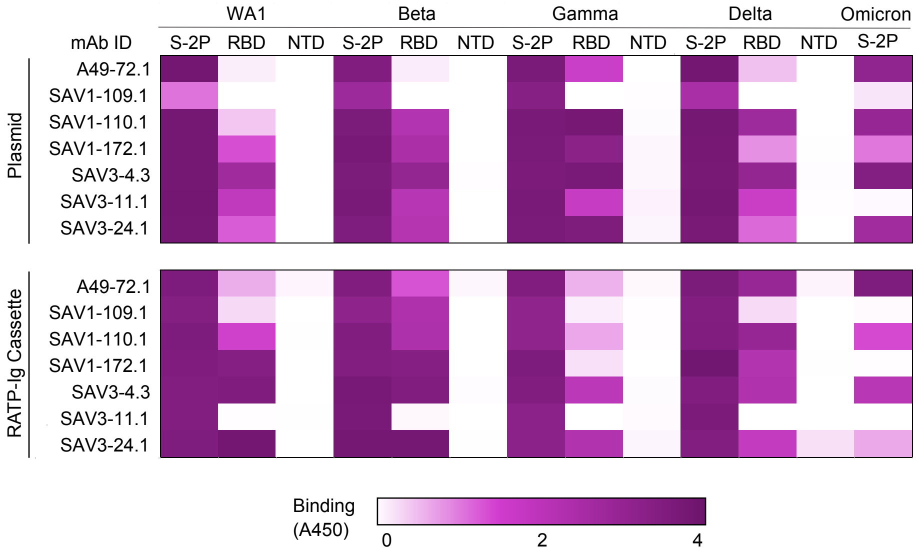

Supplementary Fig. 5: Validation of RATP-Ig screening with synthesized plasmids. Heatmaps show ELISA absorbance at 450 nm (not quantitative).

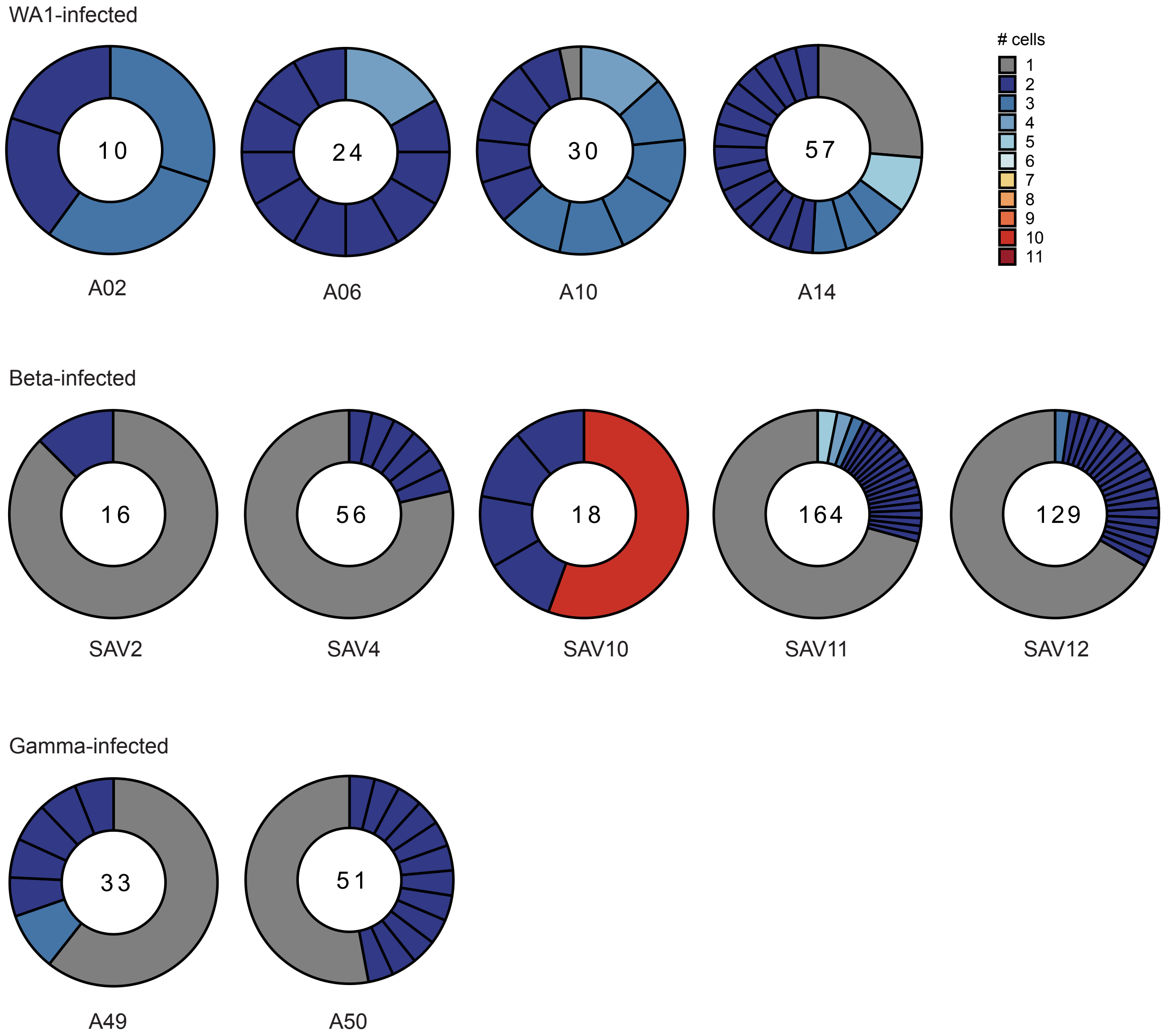

Supplementary Fig. 6: Clonal expansion in each individual obtained by BCR repertoire sequencing by 10x Genomics method. Expanded clones are colored by the number of cells in each clone as shown on the right; singleton clones are shown in gray.

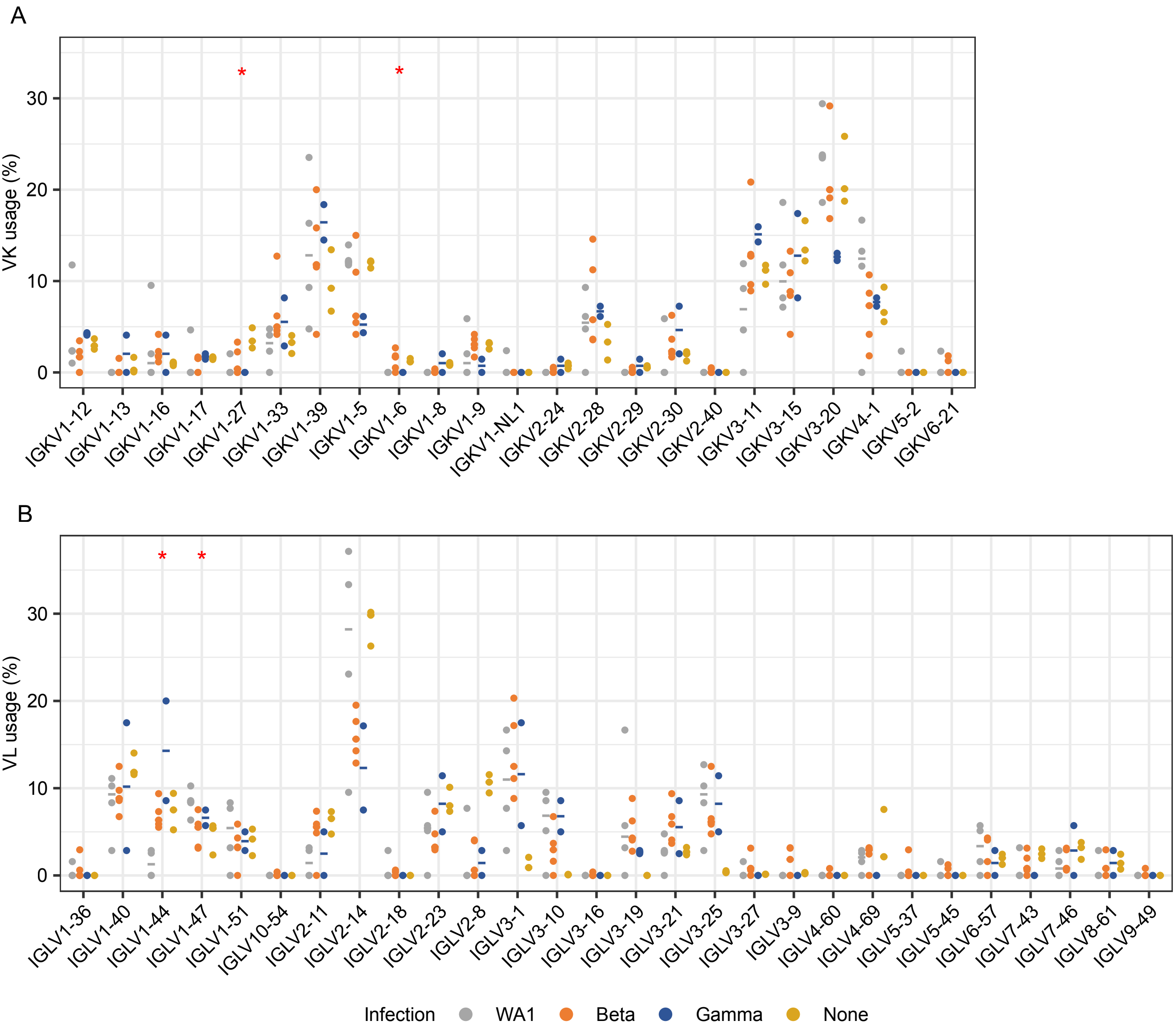

Supplementary Fig. 7: SARS-CoV-2-specific light chain V gene usage frequencies. (**A**) Kappa and (**B**) Lambda chain V gene repertoire analysis by infecting variant, with WA1, Beta and Gamma shown in grey, orange and blue, respectively, and data from pre-pandemic controls in yellow. The x-axis shows all germline genes used; the y-axis represents the percent of individual gene usage. n=349, 1826, 302, and ~1x10^{9} light chains from 4, 7, 2, and 3 individuals, for WA1-infected, Beta-infected, Gamma-infected, and historical controls, respectively. Stars indicate genes with at least one significant difference between groups; pairwise comparisons using the Dunn test are in Supplementary Table 3.

Subject ID	Infecting virus	Days after symptoms	Disease severity	Date of collection	Gender	Age
**A02**	WA1	28	Mild	Mar-20	Male	39
**A06**	WA1	34	Mild	Apr-20	Female	59
**A10**	WA1	33	Moderate	Apr-20	Female	67
**A14**	WA1	34	Mild	Apr-20	Male	27
**SAV1**	Beta	33	Severe	Jan-21	Male	60
**SAV2**	Beta	33	Mild	Jan-21	Male	35
**SAV3**	Beta	30	Mild	Jan-21	Female	58
**SAV4**	Beta	28	Mild	Jan-21	Female	30
**SAV10**	Beta	38	Mild	Feb-21	Female	43
**SAV11**	Beta	37	Mild	Feb-21	Female	52
**SAV12**	Beta	35	Mild	Feb-21	Male	44
**A49**	Gamma	24	Moderate	Jan-21	Female	53
**A50**	Gamma	17	Mild	Jan-21	Male	32

Supplementary Table 1: Details of the study cohort.

		SARS-CoV-2 Probe: WA1		Beta		Gamma		Total paired sequences by subject:
WA1-infected	A02	3/23	(13%)	3/8	(28%)	4/13	(31%)	10
	A06	11/140	(8%)	4/74	(5%)	9/62	(15%)	24
	A10	9/87	(10%)	10/46	(22%)	11/34	(32%)	30
	A14	20/205	(10%)	14/76	(18%)	23/79	(29%)	57
Beta-Infected	SAV2	2/104	(2%)	14/214	(7%)	N/A		16
	SAV4	16/328	(5%)	40/630	(6%)	N/A		56
	SAV10	6/102	(6%)	12/131	(9%)	N/A		18
	SAV11	39/645	(6%)	125/2028	(6%)	N/A		164
	SAV12	32/306	(10%)	97/1318	(7%)	N/A		129
Gamma-Infected	A49	10/129	(8%)	N/A		23/148	(16%)	33
	A50	14/89	(16%)	N/A		37/128	(29%)	51
Total paired sequences by probe:		162		319		107		

Supplementary Table 2: Sample recovery from 10x Genomics-based single cell isolation and sequencing.

Gene	Enriched Group	Median usage frequency, enriched	Depleted Group	Median usage frequency, enriched	Adjusted *P*-value
IGHV1-18	Control	7.7%	Gamma-infected	0.7%	0.043
IGHV1-18	Control	7.7%	WA1-infected	0.0%	0.007
IGHV1-46	WA1-infected	7.1%	Gamma-infected	1.7%	0.037
IGHV3-30	Gamma-infected	28.8%	Control	7.2%	0.034
IGHV3-30	WA1-infected	18.1%	Control	7.2%	0.038
IGHV4-30-2	Control	0.8%	Beta-infected	0.0%	0.016
IGHV4-30-2	Control	0.8%	WA1-infected	0.0%	0.010
IGKV1-27	Control	3.4%	Gamma-infected	0.0%	0.040
IGKV1-27	Control	3.4%	WA1-infected	0.0%	0.023
IGLV1-44	Control	7.5%	WA1-infected	1.3%	0.048
IGLV1-44	Beta-infected	6.3%	WA1-infected	1.3%	0.035
IGLV1-44	Gamma-infected	14.3%	WA1-infected	1.3%	0.017
IGLV1-47	WA1-infected	8.5%	Beta-infected	5.5%	0.027
IGLV1-47	WA1-infected	8.5%	Control	5.4%	0.041

Supplementary Table 3: Significant differences in gene-usage. For genes with a significant difference detected by the Kruskal-Wallis test (Fig. 3C and Supplementary Fig. 6), the Dunn test was used to find significant pairwise difference. P values were adjusted for multiple testing using the Benjami-Hochberg procedure. Note that *IGKV1-6* is significant in the Kruskal-Wallis test (Supplementary Fig. 6A), but none of the pairwise comparisons remain significant after correction for multiple testing.

Experiment	Marker	Fluorochrome	Clone	Supplier	Catalog number	Lot number	Dilution
**B cell repertoire sort by 10X**	IgG	FITC	G18-145	Miltenyi	130-114-001	1013930	1:40
	IgA	FITC	IS11-8E10	BD Biosciences	555786	5220201557	1:100
	CD8	BV510	RPA-T8	Biolegend	301048	B303954	1:333
	CD56	BV510	HCD56	Biolegend	318340	B318848	1:160
	CD14	BV510	M5E2	Biolegend	301842	B26341	1:100
	CD16	BUV496	3G8	BD Biosciences	612944	288806	1:200
	CD20	BUV805	2H7	BD Biosciences	564917	1085565	1:40
	IgM	CF594PE	G20-127	BD Biosciences	562539	2046723	1:77
	CD19	PC7	J3-119	Beckman Coulter	IM3628U	200152	1:77
	CD3	APCCy7	SP34-2	BD Biosciences	557757	1152687	1:50
	LIVE/DEAD Fixable Blue Dead Cell Stain			ThermoFisher	L23105	2438350	1:200
**RATP-Ig sort**	IgG	FITC	G18-145	Miltenyi	130-114-001	1013930	1:40
	IgA	FITC	IS11-8E10	BD Biosciences	555786	5220201557	1:100
	CD8	BV510	RPA-T8	Biolegend	301048	B303954	1:333
	CD56	BV510	HCD56	Biolegend	318340	B318848	1:160
	CD14	BV510	M5E2	Biolegend	301842	B26341	1:100
	CD3	BV510	OKT3	Biolegend	317332	B333939	1:100
	CD16	BUV496	3G8	BD Biosciences	612944	288806	1:200
	CD20	BUV805	2H7	BD Biosciences	564917	1085565	1:40
	IgM	CF594PE	G20-127	BD Biosciences	562539	2046723	1:77
	CD19	PC7	J3-119	Beckman Coulter	IM3628U	200152	1:77
	LIVE/DEAD Fixable Blue Dead Cell Stain			ThermoFisher	L23105	2438350	1:200
**Intracellular Cytokine Stain for T cell responses**	CD19	PE-Cy5	HIB19	BD Biosciences	302210	B263542	1:80
	CD14	BB660	M0P9	BD Biosciences	624925	0118717	1:80
	CD3	BUV395	UCHT1	BD Biosciences	563546	9303095	1:80
	CD4	BV480	SK3	BD Biosciences	566104	0058964	1:40
	CD8a	BUV805	SK1	BD Biosciences	612889	0126959	1:20
	CD45RA	BUV496	H100	BD Biosciences	750258	0118022	1:80
	CD154	PE	TRAP1	BD Biosciences	555700	0057282	1:10
	IFNg	V450	B27	BD Biosciences	560371	9274224	1:40
	IL-2	BB700	MQ1-17H12	BD Biosciences	566404	0107814	1:161
	CD16	BV570	3G8	Biolegend	302036	B292881	1:40
	CD56	BV750	5.1H11	Biolegend	362556	B299053	1:40
	CCR7	BV605	G043H7	Biolegend	353244	B322424	1:10
	CD69	APC-Fire750	FN50	Biolegend	310946	B268535	1:40
	TNF	FITC	Mab11	Biolegend	11-7349-82	2298077	1:161
	LIVE/DEAD Fixable Blue Dead Cell Stain			Invitrogen	L34962	2176884	1:200
**ELISA for mAb screening**	HRP-conjugated anti-human IgG		Polyconal	Jackson ImmunoResearch	109-035-098	154823	1:10,000
	Anti-human IgG Fc		Polyclonal	Rockland	609-1103	35325	1:20,000

Supplementary Table 4: Antibodies information.

Primer Name	Sequence
TSoligo2_polydT	CAAGCAGAAGACGGCATACGAGTTTTTTTTTTTTTTTTTTTTTTTTTTTTTT(N1:33333400)(N2:25252525)
A-tag TSO	/5Me-isodC//iisodG//iMe-isodC/ AAGCAGTGGTATCAACGCAGAGTACATArGrGrG
TSoligo2	CAAGCAGAAGACGGCATACGAG
TSO_FWD	AAGCAGTGGTATCAACGCAGAGT
IgA_REV	GAGGCTCAGCGGGAAGACCTTGGGGCTGGTCGG
IgG_REV	GCCAGGGGGAAGACCGATGGGCCCTTGGTGGA
IgK_REV	GGGTAGAAGTTATTCAGCAGGCACAC
IgL_REV	GGGTAGAAGTCACTTATGAGACACAC
CMV_FWD	CCATTGCATACGTTGTATCCATATC
CMV_TSO_REV	TATGTACTCTGCGTTGATACCACTGCTTCGACGGTGACTGCAGAAAAGAC
IgLC_FWD	ACTTCTACCCCAGAGAAGCC
IgHC_FWD	CATCGGTCTTCCCCCTG
TBGH_polyA_REV	TTAAAATTCTATGATTTCTCTCATTACTTC

Supplementary Table 5: Primer sequences for RATP-Ig and Ig sequencing.
