# Supplementary material for: Primary exposure to SARS-CoV-2 variants elicits convergent epitope specificities, immunoglobulin V gene usage and public B cell clones
Source: Nat Commun. 2022 Dec 14;13:7733. doi: 10.1038/s41467-022-35456-2 (PMC9748393; doi:10.1038/s41467-022-35456-2)
Supplement: Supplementary file 3 — Reporting Summary [file 41467_2022_35456_MOESM3_ESM.pdf]

nature portfolio

Corresponding author(s): Schramm, CA and Douek, DC

Last updated by author(s): Nov 23, 2022



# Reporting Summary

Nature Portfolio wishes to improve the reproducibility of the work that we publish. This form provides structure for consistency and transparency in reporting. For further information on Nature Portfolio policies, see our Editorial Policies and the Editorial Policy Checklist.

## Statistics

For all statistical analyses, confirm that the following items are present in the figure legend, table legend, main text, or Methods section.

| n/a | Confirmed | |
|---|---|---|
| ☐ | ☒ | The exact sample size (*n*) for each experimental group/condition, given as a discrete number and unit of measurement |
| ☐ | ☒ | A statement on whether measurements were taken from distinct samples or whether the same sample was measured repeatedly |
| ☐ | ☒ | The statistical test(s) used AND whether they are one- or two-sided<br>*Only common tests should be described solely by name; describe more complex techniques in the Methods section.* |
| ☐ | ☒ | A description of all covariates tested |
| ☐ | ☒ | A description of any assumptions or corrections, such as tests of normality and adjustment for multiple comparisons |
| ☐ | ☒ | A full description of the statistical parameters including central tendency (e.g. means) or other basic estimates (e.g. regression coefficient) AND variation (e.g. standard deviation) or associated estimates of uncertainty (e.g. confidence intervals) |
| ☐ | ☒ | For null hypothesis testing, the test statistic (e.g. *F*, *t*, *r*) with confidence intervals, effect sizes, degrees of freedom and *P* value noted<br>*Give P values as exact values whenever suitable.* |
| ☒ | ☐ | For Bayesian analysis, information on the choice of priors and Markov chain Monte Carlo settings |
| ☒ | ☐ | For hierarchical and complex designs, identification of the appropriate level for tests and full reporting of outcomes |
| ☒ | ☐ | Estimates of effect sizes (e.g. Cohen's *d*, Pearson's *r*), indicating how they were calculated |

*Our web collection on statistics for biologists contains articles on many of the points above.*

## Software and code

Policy information about availability of computer code

| | |
|---|---|
| Data collection | Biacore 8K Insights Evaluation Software v3.0.12.15655 (Cytiva)<br>BD FACSDiva Software version 9.5.1 (BD Biosciences) |
| Data analysis | FlowJo v10.7.1, v10.8.1 (BD Biosciences)<br>Graphpad Prism v.8.3.1, v9.4.0, v9.4.1<br>BALDR (https://github.com/BosingerLab/BALDR) commit 6ad99c9<br>filterBALDR (https://github.com/scharch/filterBALDR) commit 21e68b4<br>SONAR (https://github.com/scharch/SONAR) v4.2<br>Seurat v4 (https://satijablab.org/seurat)<br>usearch v9.0.2132 (https://www.drive5.com/usearch/)<br>ggseqlogo (https://omarwagih.github.io/ggseqlogo/) 4adc8f2 |

For manuscripts utilizing custom algorithms or software that are central to the research but not yet described in published literature, software must be made available to editors and reviewers. We strongly encourage code deposition in a community repository (e.g. GitHub). See the Nature Portfolio guidelines for submitting code & software for further information.

## Data

Policy information about availability of data

All manuscripts must include a data availability statement. This statement should provide the following information, where applicable:

- Accession codes, unique identifiers, or web links for publicly available datasets
- A description of any restrictions on data availability
- For clinical datasets or third party data, please ensure that the statement adheres to our policy

Raw sequencing data has been deposited in the SRA under BioProject PRJNA832903. Processed V(D)J sequences are included with the paper as a Supplementary Dataset.

## Human research participants

Policy information about studies involving human research participants and Sex and Gender in Research.

| | |
|---|---|
| Reporting on sex and gender | By sex, study include 7 females and 6 males. Gender information was not collected. No sex-based analyses were conducted due to insufficient statistical power. |
| Population characteristics | 4 donors were infected with WA1, 7 with Beta, and 2 with Gamma. Age ranged from 27-67 years. |
| Recruitment | WA1 donors were recruited at VRC early in the pandemic. Beta and Gamma donors were recruited based on clinical presentation and availability of genetic sequencing. Potential self-selection biases among people who volunteer to donate blood for these studies are not expected to have any impact on the cell-level immune responses investigated here. |
| Ethics oversight | NIH Institutional Review Board approved protocols for collecting samples |

Note that full information on the approval of the study protocol must also be provided in the manuscript.

# Field-specific reporting

Please select the one below that is the best fit for your research. If you are not sure, read the appropriate sections before making your selection.

☒ Life sciences ☐ Behavioural & social sciences ☐ Ecological, evolutionary & environmental sciences

For a reference copy of the document with all sections, see nature.com/documents/nr-reporting-summary-flat.pdf

# Life sciences study design

All studies must disclose on these points even when the disclosure is negative.

| | |
|---|---|
| Sample size | Cohort size for Beta (7 individuals) and Gamma (2 individuals) was based on availability. Cohort size for WA1 (4 individuals) was selected to be approximately equal to the other two cohorts. These cohort sizes trade-off limitations in statistical power, as noted in the manuscript, for the ability to conduct comprehensive characterizations of the immune response in each individual. |
| Data exclusions | No exclusions |
| Replication | SPR experiments were conducted in duplicate. Antibody neutralization assays were conducted in triplicate at each dilution. MSD experiments are reported as area under the curve based on four dilutions per sample, with each dilution measured one. All replicates were successful. All other assays were conducted only once. |
| Randomization | Participants were allocated to experimental groups based on infecting variant. Timing of sample collection was controlled to be in the same range (early convalescence) across all individuals. Age and sex were based on sample availability and we do not have the power to control for these. |
| Blinding | This study was not blinded, as subjects were selected based on case history with no randomization and no intervention was tested. In addition, sort probes were required to be matched to the infecting variant for the analyses described to be possible. |

# Reporting for specific materials, systems and methods

We require information from authors about some types of materials, experimental systems and methods used in many studies. Here, indicate whether each material, system or method listed is relevant to your study. If you are not sure if a list item applies to your research, read the appropriate section before selecting a response.

## Materials & experimental systems

| n/a | Involved in the study |
| --- | --- |
| ☐ | ☒ Antibodies |
| ☐ | ☒ Eukaryotic cell lines |
| ☒ | ☐ Palaeontology and archaeology |
| ☒ | ☐ Animals and other organisms |
| ☒ | ☐ Clinical data |
| ☒ | ☐ Dual use research of concern |

## Methods

| n/a | Involved in the study |
| --- | --- |
| ☒ | ☐ ChIP-seq |
| ☐ | ☒ Flow cytometry |
| ☒ | ☐ MRI-based neuroimaging |

## Antibodies

Antibodies used

B cell repertoire sort antibodies
Marker Fluorochrome clone Supplier catalog # lot #
IgG FITC G18-145 Miltenyi 130-114-001 1013930
IgA FITC IS11-8E10 BD Biosciences 555786 5220201557
CD8 BV510 RPA-T8 Biolegend 301048 B303954
CD56 BV510 HCD56 Biolegend 318340 B318848
CD14 BV510 M5E2 Biolegend 301842 B26341
CD16 BUV496 3G8 BD Biosciences 612944 288806
CD20 BUV805 2H7 BD Biosciences 564917 1085565
IgM CF594PE G20-127 BD Biosciences 562539 2046723
CD19 PC7 J3-119 Beckman Coulter IM3628U 200152
CD3 APCCy7 SP34-2 BD Biosciences 557757 1152687
LD UV blue ThermoFisher L23105 2438350

RATP-Ig sort antibodies
Marker Fluorochrome clone Supplier catalog # lot #
IgG FITC G18-145 Miltenyi 130-114-001 1013930
IgA FITC IS11-8E10 BD Biosciences 555786 5220201557
CD8 BV510 RPA-T8 Biolegend 301048 B303954
CD56 BV510 HCD56 Biolegend 318340 B318848
CD14 BV510 M5E2 Biolegend 301842 B26341
CD3 BV510 OKT3 Biolegend 317332 B333939
CD16 BUV496 3G8 BD Biosciences 612944 288806
CD20 BUV805 2H7 BD Biosciences 564917 1085565
IgM CF594PE G20-127 BD Biosciences 562539 2046723
CD19 PC7 J3-119 Beckman Coulter IM3628U 200152
LIVE/DEAD Fixable Blue Dead Cell Stain ThermoFisher L23105 2438350

ICS Antibodies
Marker Fluorochrome clone Supplier catalog # lot #
CD19 PE-Cy5 HIB19 BD Biosciences 302210 B263542
CD14 BB660 M0P9 BD Biosciences 624925 0118717
CD3 BUV395 UCHT1 BD Biosciences 563546 9303095
CD4 BV480 SK3 BD Biosciences 566104 0058964
CD8a BUV805 SK1 BD Biosciences 612889 0126959
CD45RA BUV496 H100 BD Biosciences 750258 0118022
CD154 PE TRAP1 BD Biosciences 555700 0057282
IFNg V450 B27 BD Biosciences 560371 9274224
IL-2 BB700 MQ1-17H12 BD Biosciences 566404 0107814
CD16 BV570 3G8 Biolegend 302036 B292881
CD56 BV750 5.1H11 Biolegend 362556 B299053
CCR7 BV605 G043H7 Biolegend 353244 B322424
CD69 APC-Fire750 FN50 Biolegend 310946 B268535
TNF FITC Mab11 Biolegend 11-7349-82 2298077
LIVE/DEAD Fixable Blue Dead Cell Stain Invitrogen L34962 2176884

ELISA for mAb screening
Marker Fluorochrome clone Supplier catalog # lot #
HRP-conjugated anti-human IgG n/a Jackson ImmunoResearch 109-035-098 154823
Anti-human IgG Fc n/a Rockland 609-1103 42050

| | |
|---|---|
| Validation | All reagent antibodies were purchased from commercial sources and were not validated further. |

## Eukaryotic cell lines

Policy information about cell lines and Sex and Gender in Research

| | |
|---|---|
| Cell line source(s) | Expi293F cells (ThermoFisher Scientific) |
| Authentication | No cell lines were authenticated |
| Mycoplasma contamination | Cell lines were not tested for mycoplasma contamination |
| Commonly misidentified lines (See ICLAC register) | None used |

## Flow Cytometry

### Plots

Confirm that:

- [x] The axis labels state the marker and fluorochrome used (e.g. CD4-FITC).
- [x] The axis scales are clearly visible. Include numbers along axes only for bottom left plot of group (a 'group' is an analysis of identical markers).
- [x] All plots are contour plots with outliers or pseudocolor plots.
- [x] A numerical value for number of cells or percentage (with statistics) is provided.

### Methodology

Sample preparation: Cryopreserved PBMC were thawed into pre-warmed R10 media (RPMI 1640, 10% FBS, 2 mM L-glutamine, 100 U/ml penicillin, and 100 μg/ml streptomycin) containing DNase.

Instrument: For B cell sort: BD FACSAria II instrument (BD Biosciences); for ICS: BD FACSymphony (BD Biosciences)

Software: FlowJo software (version 10.7.1 or 10.8.1)

Cell population abundance:

For B cell repertoire sort
Sample ID Probes used Number of B cells sorted
SAV2 "WT RBDWT S-2PWT NTD" 104
SAV4 328
SAV10 102
SAV11 645
SAV12 306
A49 129
A50 89

SAV2 "Beta RBDBeta S-2PBeta NTD" 214
SAV4 630
SAV10 131
SAV11 2028
SAV12 1318

A49 "Gamma RBDGamma S-2PGamma NTD" 148
A50 128
A02 "WT RBDWT S-2PWT NTD" 23
A06 140
A10 87
A14 205
A02 "Beta RBDBeta S-2PBeta NTD" 8
A06 74
A10 46
A14 76

A02 "Gamma RBDGamma S-2PGamma NTD" 13
A06 62
A10 34
A14 79

For RATP-Ig

SAV1 WT and Beta RBD, S-2P and NTD 177
SAV3 186
A49 137

The purity of the cells was determined by the flow cytometry using the gating strategy provided.

| | |
|---|---|
| Gating strategy | Gating strategies and gate boundaries are shown as flow plots in the Extended Data. Briefly, for B cell sorting and analysis, cells were gated on FSC(A)xSSC(A) -> FSC(H)xFSC(A) -> CD3-LD- -> CD8-CD56-CD14-CD16- -> CD19+CD20+ -> IgG/IgA+ IgM- -> probe+. For ICS, memory T cells were gated on Timex SSC -> FSC(A)xSSC(A) -> FSC(H)xFSC(A) -> CD19-CD14-CD16-CD56- -> LD-CD3+ -> CD4+ or CD8+ -> CD45RA+CCR7- or CD45RA-CCR7- or CD45RA-CCR7+ -> CD4+ CD69+CD154+ or CD8+ CD69+TNF+ or IL2+ or IFNg+ |

☒ Tick this box to confirm that a figure exemplifying the gating strategy is provided in the Supplementary Information.
