# Supplementary material for: Primary exposure to SARS-CoV-2 variants elicits convergent epitope specificities, immunoglobulin V gene usage and public B cell clones
Source: Nat Commun. 2022 Dec 14;13:7733. doi: 10.1038/s41467-022-35456-2 (PMC9748393; doi:10.1038/s41467-022-35456-2)
Supplement: Supplementary file 4 — Description of Additional Supplementary Files [file 41467_2022_35456_MOESM4_ESM.docx]

**Description of Additional Supplementary Files**

File Name: Supplementary Data 1

Description: Sequences of peptides included in Spike pools A and B used for T cell stimulation.

File Name: Supplementary Data 2

Description: Sequences of peptides included in selected peptide pools for each variant used for T cell stimulation.

File Name: Supplementary Data 3

Description: Complete RATP-Ig ELISA results for SAV1. Values are reported as absorbance at 450nm wavelength.

File Name: Supplementary Data 4

Description: Complete RATP-Ig ELISA results for SAV3. Values are reported as absorbance at 450nm wavelength.

File Name: Supplementary Data 5

Description: Complete RATP-Ig ELISA results for A49. Values are reported as absorbance at 450nm wavelength.

File Name: Supplementary Data 6

Description: BCR sequences for all clones obtained by RATP-Ig or 10x Genomics.
